# Angiotensin-(1-7) and Its Effects in the Kidney

**DOI:** 10.1100/tsw.2009.70

**Published:** 2009-06-30

**Authors:** Marc Dilauro, Kevin D. Burns

**Affiliations:** Departments of Medicine and Cellular and Molecular Medicine, Ottawa Hospital, University of Ottawa, Canada

**Keywords:** renin-angiotensin system, angiotensin, ACE2, Mas receptor, proximal tubule, glomerulus

## Abstract

Angiotensin-(1-7) (Ang-[1-7]) is a heptapeptide member of the renin-angiotensin system (RAS), and acts as a vasodilator and antagonist of angiotensin II (Ang II) in the vasculature. The role of Ang-(1-7) in regulating kidney function is not well understood. Within the kidneys, Ang-(1-7) is generated by angiotensin-converting enzyme 2 (ACE2)–mediated degradation of Ang II, sequential cleavage of the precursor angiotensin I (Ang I) by ACE2 and ACE, or the actions of brush-border membrane peptidases on Ang I. Ang-(1-7) mediates its effects via binding to kidney Mas receptors, although some actions may occur via Ang II AT_1_ or AT_2_ receptors. *In vitro* studies suggest that Ang-(1-7) is an intrarenal vasodilator. Ang-(1-7) has been reported to induce either natriuresis/diuresis or sodium and water retention, via modulation of sodium transporters in the proximal tubule and loop of Henle, and collecting duct water transport. In the proximal tubule, Ang-(1-7) antagonizes growth-promoting signaling pathways via activation of a protein tyrosine phosphatase, whereas in mesangial cells, Ang-(1-7) stimulates cell growth via activation of mitogen-activated protein kinases. The phenotype of the Mas gene knockout mouse suggests that Ang-(1-7)–signaling events exert cardiovascular protection by regulating blood pressure, and by limiting production of reactive oxygen species and extracellular matrix proteins. Ang-(1-7) also protects against renal injury in the renal wrap hypertension model, independent of effects on blood pressure. In diabetic nephropathy, however, the role of Ang-(1-7) on disease progression remains unclear. In summary, Ang-(1-7) and its receptor Mas have emerged as important components of the intrarenal RAS. The signaling and downstream effects of Ang-(1-7) in the kidney are complex and appear to be cell specific. The body of evidence suggests that Ang-(1-7) is protective against endothelial dysfunction or Ang II–stimulated proximal tubular injury, although the overall effects on glomerular function require further study.

## INTRODUCTION

Angiotensin-(1-7) (Ang-[1-7]) is a biologically active heptapeptide that has been postulated to counterbalance the physiological actions of angiotensin II (Ang II) within the renin-angiotensin system (RAS)[1]. In recent years, several key findings have increased our understanding of the classical RAS and the biological significance of Ang-(1-7). For instance, angiotensin-converting enzyme 2 (ACE2) was identified as an important Ang-(1-7)–forming enzyme[2,3], and the G protein–coupled receptor Mas was discovered to be an endogenous binding site for Ang-(1-7)[4]. To date, research conducted on Ang-(1-7) has largely focused on its cardiovascular actions. However, the kidney has emerged as an important target for the actions of Ang-(1-7). In this review, we highlight recent advances in our understanding of intrarenal Ang-(1-7), and its role in kidney physiology and pathophysiology.

## FORMATION AND METABOLISM OF INTRARENAL ANG-(1-7)

Ang-(1-7) is generated in the vascular endothelium[5]. Thus, in human endothelial cells, Santos et al. demonstrated local production of Ang-(1-7) from angiotensin I (Ang I) or Ang II[5]. Endothelial production of Ang-(1-7) may contribute to circulating levels and, indeed, under physiological conditions, plasma concentrations of Ang-(1-7) are in the picomolar range, comparable to the plasma levels of Ang II[6,7,8]. Evidence for the generation of intrarenal Ang-(1-7) derives from several observations. First, intrarenal levels of Ang-(1-7) are relatively high and comparable to levels of Ang II[8]. In Sprague-Dawley rats, Ang-(1-7) and Ang II are present at ~300 and ~800 fmol/g kidney tissue, respectively[8]. Second, the enzymes necessary for Ang-(1-7) formation are abundant in the kidney and have also been detected in urine[9]. Third, Ang-(1-7) is detectable in urine from human subjects[10]. Indeed, Ferrario et al. reported that untreated essential hypertensive subjects exhibit lower urinary concentrations than normotensive controls, suggesting that Ang-(1-7) may play a role in the regulation of blood pressure[10].

In the kidney, Ang-(1-7) appears to be generated from its precursor Ang I or by the degradation of Ang II[11,12]. This process is mediated by various protease enzymes including neprilysin (NEP), thimet oligopeptidase (TOP), and prolyl oligopeptidase (POP) (Fig. 1), which are located either on brush-border membranes or in the cytoplasm[13,14]. Aside from the proximal tubule, however, the contribution of more distal nephron segments to the formation of Ang-(1-7) is unclear.

ACE2 is a homologue of angiotensin-converting enzyme (ACE)[2,3] that degrades Ang II to Ang-(1-7) and converts Ang I to angiotensin-(1-9), which in turn may be cleaved by ACE to Ang-(1-7)[15]. Of importance, the enzymatic activity of ACE2 is not blocked by conventional ACE inhibitors[15]. ACE2 is highly expressed in the kidney and colocalizes immunohistochemically with Ang-(1-7) to the proximal tubule[16]. In rat kidney, ACE2 mRNA has been detected in all nephron segments except the thick ascending limb of the loop of Henle, with relatively high levels in the proximal straight tubule[17].

In isolated rat proximal tubular segments, Li et al. demonstrated that Ang-(1-7) is generated from Ang I in an ACE2-dependent manner[17]. However, incubation with Ang II or luminal perfusion of Ang II did not result in the generation of Ang-(1-7)[17]. Conversely, studies in rat kidney cortex and isolated sheep proximal tubules have shown that Ang-(1-7) is primarily generated via the ACE2-dependent degradation of Ang II[18,19]. Importantly, Shaltout et al. were unable to detect processing of Ang I by ACE2 in sheep proximal tubules following inhibition of ACE and neprilysin[18]. Indeed, i*n vitro* kinetic data indicate that ACE2 catalytic efficiency is ~400-fold higher with Ang II as a substrate than with Ang I[15]. Although it is difficult to reconcile these data, it is possible that differences in experimental preparations (for example, microdissected proximal tubules vs. proximal tubular preparations) may account for the discrepancies in proximal tubular processing of Ang I and Ang II. In this regard, in the C57BL/6 mouse with ACE2 gene deletion, intrarenal levels of Ang II have been found to be increased, suggesting impaired Ang II processing when ACE2 is absent[20]. Nonetheless, further studies are required to determine the relative contributions of ACE2- and non-ACE2–dependent pathways to the formation of Ang-(1-7) in proximal tubule.

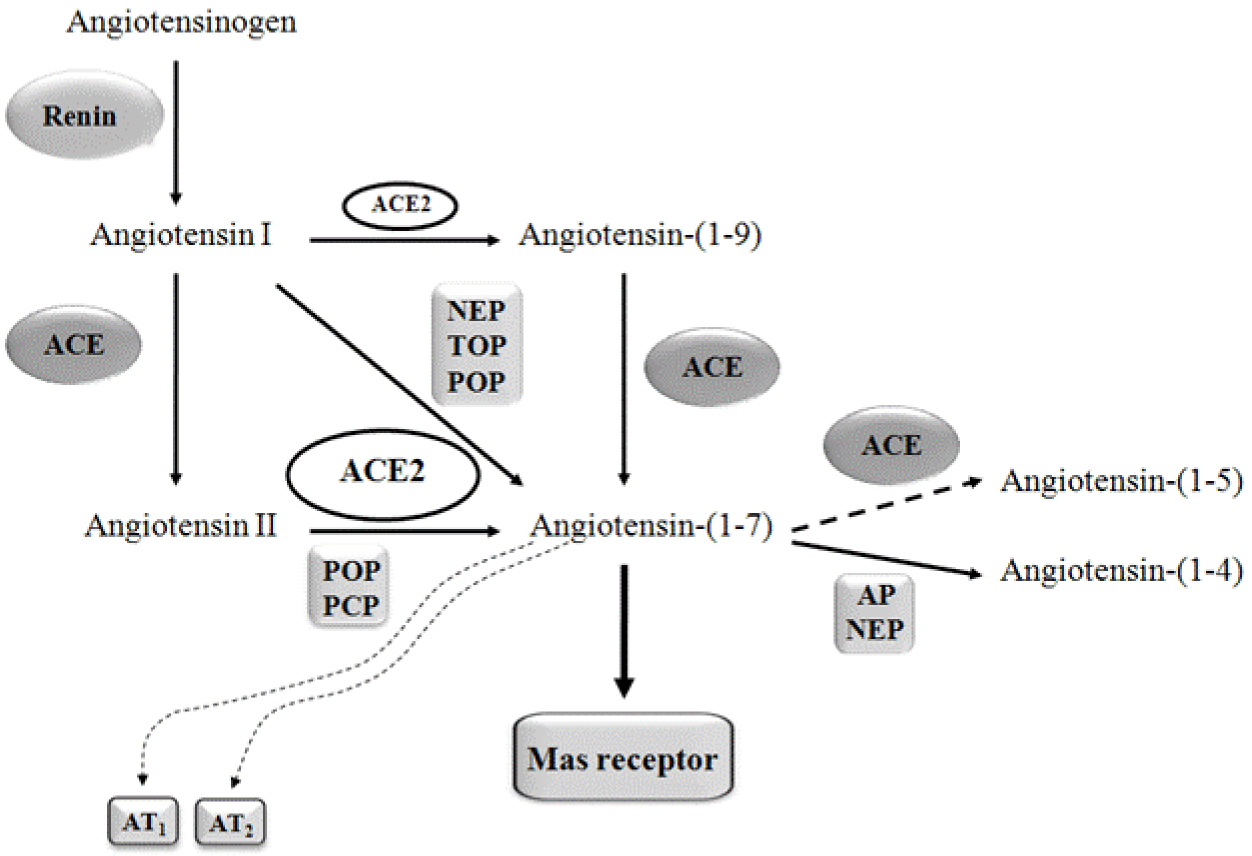

**FIGURE 1**. Pathways for formation and degradation of Ang-(1-7) in the kidney. Angiotensinogen is cleaved by renin to form the decapeptide Ang I, which is acted on by ACE to form the octapeptide Ang II. Ang-(1-7) is primarily generated via the ACE2-dependent metabolism of Ang II. ACE2 can also convert Ang I to Ang-(1-9), albeit at a lower efficiency, which can be metabolized by ACE to yield Ang-(1-7). Prolyl oligopeptidase (POP) and prolyl carboxypeptidase (PCP) degrade Ang II to Ang-(1-7). Alternatively, the precursor Ang I can be directly converted to Ang-(1-7) by the action of the following enzymes: neprilysin (NEP), thimet oligopeptidase (TOP), and POP. The physiological actions of Ang-(1-7) are mediated primarily by Mas receptors and to a lesser extent by $AT_1$ and $AT_2$ receptors (small dashed arrows). Ang-(1-7) is metabolized primarily by aminopeptidase (AP) and NEP, which convert Ang-(1-7) to the inactive peptide Ang-(1-4). ACE can also convert Ang-(1-7) to Ang-(1-5) (large dashed arrow), which is considered inactive.

ACE2 protein expression has also been detected in the glomerulus. Ye et al. reported that in the normal mouse glomerulus, ACE2 immunofluorescence colocalizes with podocyte and mesangial cell markers, but not endothelial cell markers[21]. In human kidney, however, ACE2 expression has been observed in the renal capillary endothelium[22]. Electron microscopy immunogold labeling has also localized ACE2 to podocytes[21].

In immortalized mouse podocytes, Velez et al. showed that Ang-(1-7) was the predominant peptide product after incubation with Ang II or Ang I[23]. In the presence of Ang II as a substrate, preincubation of podocytes with the ACE2 inhibitor DX-600 did not alter levels of Ang-(1-7). However, levels of Ang-(1-7) were close to the lower limit of detection and therefore could not be precisely quantified. In contrast, when podocytes were incubated with Ang I, the formation of Ang-(1-7) was significantly blocked by the NEP inhibitor thiorphan. These data suggest that in podocytes, NEP plays a key role in Ang-(1-7) generation. Although ACE2 may convert Ang II to Ang-(1-7) in podocytes, the relative contribution of ACE2 to total Ang-(1-7) formation remains unclear.

Using rat renal brush-border membrane preparations, Allred et al. reported that Ang-(1-7) is rapidly hydrolyzed to angiotensin-(1-4) (Ang-[1-4]) and smaller peptide fragments[24]. Incubation with the aminopeptidase inhibitor amastatin attenuates the hydrolysis of Ang-(1-7). Similarly, combined incubation with amastatin and the NEP inhibitor SCH-39370 abolishes the formation of Ang-(1-4) and increases the half-life of Ang-(1-7). In addition to its metabolism to Ang-(1-4), ACE has been shown to degrade Ang-(1-7) to angiotensin-(1-5)[25,26].

## KIDNEY RECEPTORS FOR ANG-(1-7)

In the mouse, deletion of the Mas proto-oncogene abolishes the intrarenal binding and functional responses to Ang-(1-7)[4]. Radioligand binding studies indicate that Mas-transfected Chinese hamster ovary (CHO) cells bind Ang-(1-7) with high affinity ($K_d$ 0.83 n*M*), with displacement by the Ang-(1-7) receptor antagonist A-779[4]. These findings support a role for the G protein–coupled Mas receptor in mediating the renal signaling effects of Ang-(1-7). Ang-(1-7) may also bind to Ang II $AT_1$ and $AT_2$ receptors, although radioligand binding assays suggest that these are low-affinity interactions[27]. Nonetheless, this raises the possibility that certain physiological effects of intrarenal Ang-(1-7) may be mediated by non-Mas receptors.

Using fluorescent *in situ* hybridization, Alenina et al. reported that Mas mRNA is abundant in mouse renal cortex[28]. Mas mRNA has also been detected in rat renal cortex and in primary cultures of rat proximal tubular cells by reverse transcription polymerase chain reaction[29]. By immunocytochemistry, the Mas receptor is expressed in the mouse afferent arteriole and in tubular epithelium, primarily on the apical surface[30]. Finally, in cultured human proximal tubular cells and human mesangial cells, Mas protein expression has been detected by immunoblot[31].

Megalin is a single-transmembrane domain receptor that belongs to the low-density lipoprotein receptor family[32]. Megalin is expressed in the brush border of the proximal tubule, where it mediates the nonspecific uptake of peptides that escape through the glomerular filtration barrier[32]. Studies in mouse renal brush-border membrane vesicles reveal that the megalin receptor binds and internalizes Ang-(1-7)[33]. The biological significance of megalin-mediated uptake of Ang-(1-7) in the kidney is unclear. However, these findings suggest that megalin may serve as a major uptake pathway for Ang-(1-7) in the proximal tubule.

## SIGNALING PATHWAYS AND FUNCTIONS OF ANG-(1-7) IN THE KIDNEY

### Regulation of Renal Hemodynamics by Ang-(1-7)

In the nonrenal vasculature, Ang-(1-7) exerts a vasodilatory effect that involves increased production of nitric oxide (NO), prostaglandins, or endothelium-dependent hyperpolarizing relaxing factor[34,35,36]. Sampaio et al. revealed the molecular basis for the NO-releasing activity of Ang-(1-7)[37]. In human aortic endothelial cells and CHO cells stably transfected with Mas cDNA, Ang-(1-7) induced NO release by stimulating endothelial nitric oxide synthase (eNOS) and Akt phosphorylation. These effects were blocked by the Ang-(1-7) receptor antagonist A-779, suggesting involvement of Mas receptor signaling pathways.

The role of Ang-(1-7) in the regulation of renal hemodynamics is incompletely understood and data are conflicting. Van der Wouden et al. assessed the *in vitro* and *in vivo* effects of Ang-(1-7) in rat renal vasculature[38]. Although Ang-(1-7) alone did not affect vascular function, it prevented Ang II–induced vasoconstriction of isolated renal arteries *in vitro*. This is in agreement with studies by Ren et al., who showed that Ang-(1-7) causes afferent arteriolar dilatation, mediated by production of NO[39]. In anesthetized rats *in vivo*, however, Ang-(1-7) did not affect Ang II–induced afferent and efferent arteriolar constriction, nor did it alter Ang II–induced renal blood flow reduction in freely moving rats[38]. Similarly, in sodium-replete Wistar rats, Handa et al. demonstrated that Ang-(1-7) did not affect the decrease in renal blood flow induced by intrarenal bolus injection of Ang II[40]. These findings highlight the difficulty in eliciting renal hemodynamic effects of Ang-(1-7) *in vivo*. In this regard, since renal blood flow is regulated by numerous vasoconstrictor and vasodilator influences, it is possible that the vasodilatory effects of Ang-(1-7) may be masked *in vivo*[41].

Other studies support a regulatory role for Ang-(1-7) in the renal vasculature. Benter et al. showed that infusion of Ang-(1-7) into spontaneously hypertensive rats (SHR) normalizes systolic blood pressure[42]. In Wistar-Kyoto rats and SHR, Ang-(1-7) increases renal blood flow and inhibits Ang II pressor responses[37,43]. The latter effect is blocked by antagonism of the Mas receptor, cyclo-oxygenase inhibition, or NOS inhibition, suggesting a role for Mas-mediated release of prostaglandins and NO in the vasodilatory response to Ang-(1-7).

## Regulation of Renal Tubular Transport by Ang-(1-7)

The role of Ang-(1-7) in the regulation of salt and water excretion has been the subject of several studies and, as with hemodynamic responses, the data have been difficult to reconcile. In anesthetized rats, administration of Ang-(1-7) increases urinary flow rate and sodium excretion, an effect abolished by A-779[40]. Similarly, intrarenal infusion of Ang-(1-7) in dogs increases urinary excretion of water and sodium, although this is partially blocked by the $AT_1$ receptor antagonist EXP 3174, but not by the $AT_2$ receptor antagonist PD123319, suggesting a role for Ang-(1-7)–mediated signaling via the $AT_1$ receptor[44].

Ang-(1-7) may limit transcellular sodium transport by regulating the activity of transporters in the proximal tubule. In cultured rabbit proximal tubular cells, Ang-(1-7) inhibits sodium flux, an effect associated with activation of phospholipase $A_2$[45]. In isolated rat proximal tubules, Ang-(1-7) inhibits the ouabain-sensitive $Na^+$-$K^+$-ATPase[40]. Furthermore, in isolated pig kidney inner cortical membranes, Ang-(1-7) inhibits $Na^+$-ATPase activity, an effect that is reversed by $AT_2$ receptor antagonism[46]. On the other hand, in isolated rat proximal straight tubules, Garcia and Garvin demonstrated that Ang-(1-7) acts through $AT_1$ receptors to exert a biphasic effect on fluid absorption[47]. At low concentrations ($10^{-12}$ *M*), Ang-(1-7) increases fluid absorption ($J_v$), whereas at high concentrations ($10^{-8}$ *M*), it inhibits $J_v$. Taken together, these data suggest that Ang-(1-7) causes natriuresis and diuresis via activation of Mas receptors, although there may also be involvement of $AT_1$ and $AT_2$ receptors.

In contrast, in water-loaded rats, Santos et al. demonstrated that infusion of Ang-(1-7) decreased urine volume, an effect reversed by Mas receptor antagonism[48]. This finding suggests that the antidiuretic effect of Ang-(1-7) is mediated, at least in part, by the Mas receptor. Joyner et al. reported that administration of Ang-(1-7) to Sprague-Dawley rats results in antidiuresis associated with up-regulation of renal aquaporin-1[49]. Chronic administration of the Mas receptor antagonist A-779 to either normal rats or SHR causes diuresis and natriuresis[50]. In virgin female rats, administration of A-779 significantly increases urine volume, suggesting that Ang-(1-7) exerts an antidiuretic effect[51]. Consistent with the previous findings, transgenic rats (TGR[A1-7]3292) that overexpress an Ang-(1-7) fusion protein, which leads to elevated plasma concentrations of Ang-(1-7), display a reduction in basal urinary flow in comparison to control rats[52]. Importantly, no significant differences in glomerular filtration rate, urinary sodium and potassium excretion, or circulating levels of vasopressin were observed between TGR(A1-7)3292 and control rats. Therefore, increased circulating levels of Ang-(1-7) appear to cause an antidiuretic effect that is independent of vasopressin release.

Aside from regulating salt and water excretion in the proximal tubule, Ang-(1-7) may modulate transport in other nephron segments. In anesthetized Munich-Wistar rats, renal micropuncture experiments revealed that Ang-(1-7) ($10^{-8}$ *M*) increased fluid, potassium, and sodium reabsorption in the loop of Henle[53]. This response was abolished by $AT_1$ receptor antagonism. Notably, infusion of Ang-(1-7) at physiological concentrations ($10^{-12}$ to $10^{-8}$ *M*) did not affect tubular fluid reabsorption in the proximal convoluted or distal tubule[53]. These studies therefore suggest that Ang-(1-7) increases reabsorption in the loop of Henle by an $AT_1$ receptor–mediated mechanism.

In isolated perfused rat inner medullary collecting duct (IMCD), Santos et al. demonstrated that Ang-(1-7) ($10^{-9}$ *M*) enhances water transport, an effect that is mediated by the Mas receptor[48]. In isolated rat

IMCD cell suspensions, Magaldi et al. showed that Ang-(1–7) ($10^{-9}$ *M*) increases cAMP production[54]. This response was attenuated by Mas receptor antagonism and by pharmacological blockade of the vasopressin V2 receptor. These data provide evidence that Ang-(1-7) regulates water transport in the IMCD, possibly via a cross-talk mechanism between the Mas receptor and the vasopressin system, involving activation of adenylate cyclase.

An alternate approach to studying the effects of Ang-(1-7) on kidney water handling has been to use the nonpeptide Ang-(1-7) agonist AVE-0991. In water-loaded mice, Pinheiro et al. showed that AVE-0991 caused a decrease in urine volume, associated with an increase in urine osmolality[55]. The antidiuretic effect of AVE-0991 was completely blocked by A-779, suggesting that AVE-0991 mimics the renal actions of Ang-(1-7) by binding to the Mas receptor. However, the antidiuretic effect of AVE-0991 is also blocked by $AT_1$ and $AT_2$ receptor antagonism. The data therefore indicate that cross-talk mechanisms may exist between the Mas, $AT_1$, and $AT_2$ receptors that regulate water transport in the kidney.

Discrepancies between the studies regarding the role of Ang-(1-7) in the regulation of renal hemodynamics as well as salt and water excretion may be explained by differences in experimental design and use of *in vitro* vs. *in vivo* preparations. Due to the rapid degradation of Ang-(1-7) in plasma, *in vivo* studies that involve infusion of Ang-(1-7) may be associated with lower plasma levels than the concentrations that are utilized *in vitro*[38,56]. Moreover, anesthesia may complicate the interpretation of experimental findings, since it may induce significant effects on renal hemodynamics, including the lowering of vascular resistance and blood pressure[57]. Finally, it appears that although Mas receptors mediate many of the effects of Ang-(1-7) along the nephron, Ang II receptors may also be involved and could influence the net tubular effects. It is therefore evident that further studies are required to unravel the effects of Ang-(1-7) on tubular transport, since current information is difficult to reconcile into a comprehensive model.

## Intrarenal Ang-(1-7) and Cell Growth Pathways

In addition to its effects on renal hemodynamics and tubular transport, Ang-(1-7) may regulate cell growth in the kidney. In rat proximal tubular cells, Ang-(1-7) inhibits Ang II–stimulated phosphorylation of three mitogen-activated protein kinases (MAPK) (p38, extracellular signal-related kinase [ERK1/2], and c-Jun N-terminal kinase [JNK]), an effect that is reversed by A-779[29]. Ang-(1-7) also partially inhibits Ang II–stimulated production of the profibrotic cytokine transforming growth factor-β1 (TGF-β1). The inhibitory effect of Ang-(1-7) on MAPK phosphorylation in the proximal tubule involves activation of a protein tyrosine phosphatase. In renal epithelial LLC-PK cells, Gava et al. demonstrated that Ang-(1-7) binds the Mas receptor and blocks high glucose-stimulated phosphorylation of p38 MAPK, an effect that is associated with activation of Src-homology 2–containing protein-tyrosine phosphatase-1 (SHP-1)[58]. Ang-(1-7) also inhibits high glucose-stimulated cell protein synthesis and prevents the stimulatory effect of glucose on TGF-β1. These findings are consistent with the reported growth inhibitory properties of Ang-(1-7) on cardiac tissue. For instance, infusion of Ang-(1-7) into Sprague-Dawley rats protects against Ang II–induced cardiac myocyte hypertrophy and interstitial fibrosis[59]. Pretreatment of adult rat cardiac fibroblasts with Ang-(1-7) inhibits Ang II–induced increases in collagen synthesis and mRNA expression of growth factors[60]. Furthermore, in a rat deoxycorticosterone (DOCA)-salt model of hypertension, infusion of Ang-(1-7) prevented myocardial and perivascular fibrosis by inhibiting collagen deposition[61]. Ang-(1-7) has also been shown to inhibit the growth of cultured neonatal rat myocytes via activation of the Mas receptor[62].

The inhibitory effects of Ang-(1-7) on Ang II–mediated signaling events may involve other pathways besides direct mediation via the Mas receptor. For example, cross-talk may exist between Mas, $AT_1$, $AT_2$, or bradykinin B2 receptors. In this regard, Kostenis et al. showed that the Mas receptor can hetero-oligomerize with the $AT_1$ receptor and inhibit the intracellular calcium mobilization effect of Ang II[63].

In contrast to these growth inhibitory effects, in mesangial cells, Ang-(1-7) may stimulate growth. Zimpelmann and Burns showed that Ang-(1-7) increased MAPK phosphorylation in cultured human mesangial cells, associated with stimulation of DNA synthesis, arachidonic acid release, and production of TGF-β1 and extracellular matrix (ECM) proteins[31]. These cells were shown to express the Mas receptor and that the growth stimulatory effects of Ang-(1-7) were inhibited by A-779, but not by $AT_1$ or $AT_2$ receptor antagonism, and were dependent on upstream activation of p38 MAPK. Although Mas siRNA knockdown was not performed in these studies, the results strongly suggest that binding of Ang-(1-7) to the Mas receptor stimulates mesangial cell growth responses. In mouse bone marrow–derived dendritic cells, Ang-(1-7) increases Ang II–mediated ERK1/2 phosphorylation, an effect blocked by A-779[64]. Ang-(1-7) also potently stimulates proliferation of human cord blood hematopoietic progenitor cells, both *in vitro* and *in vivo*[65].

In summary, in the proximal tubule, Ang-(1-7) displays growth inhibitory properties and antagonizes the effects of Ang II and high glucose, whereas in mesangial cells, it appears to stimulate cell growth pathways (Fig. 2). Therefore, the overall effect of Ang-(1-7) on kidney cell growth and function is unclear and awaits further studies, including those directed at other glomerular cells and tubular segments.

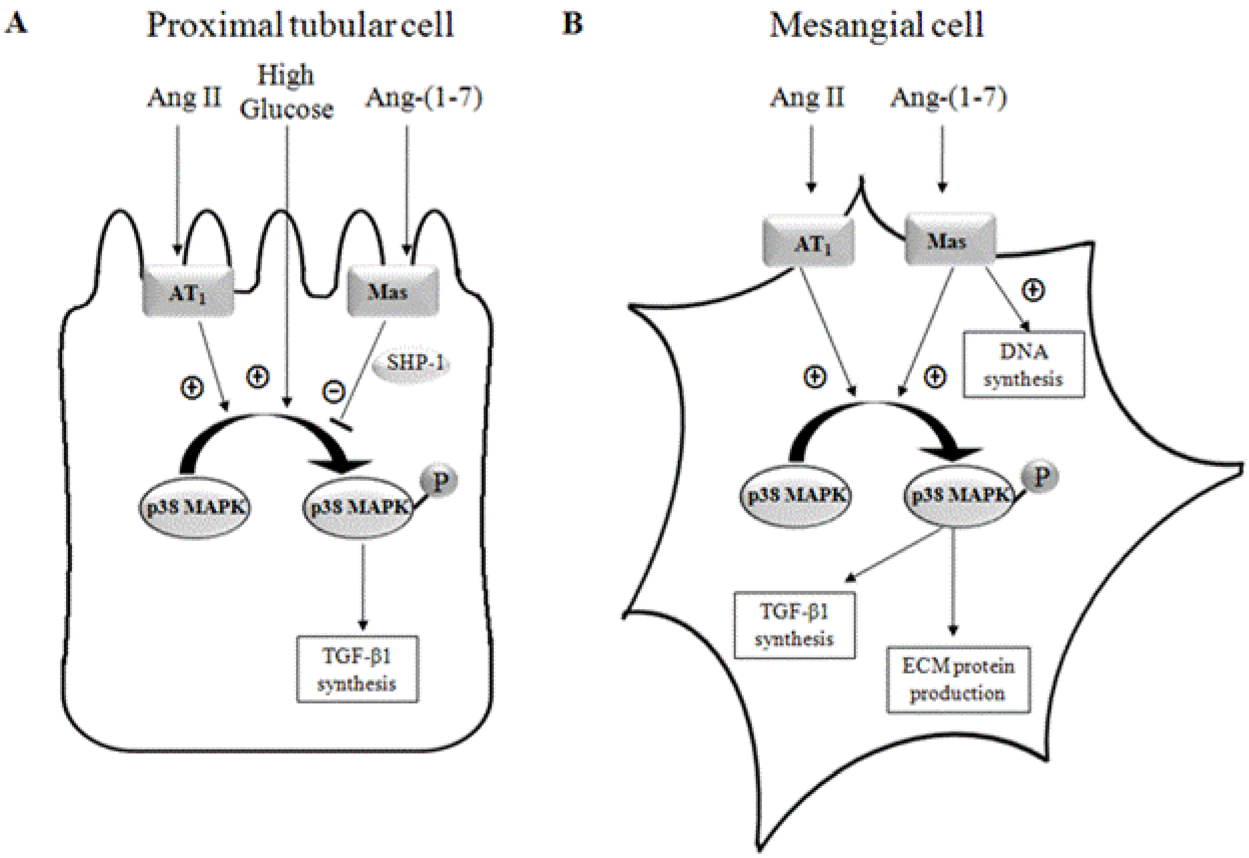

**FIGURE 2.** Signaling pathways for Ang-(1-7) in proximal tubule and mesangial cells. (A) In proximal tubular cells, Ang-(1-7) inhibits Ang II– or high glucose–stimulated phosphorylation of p38 MAPK via activation of SHP-1. Inhibition of p38 MAPK results in decreased synthesis of the profibrotic cytokine TGF-β1. (B) In contrast, in mesangial cells, Ang-(1-7) stimulates cell growth pathways. Ang-(1-7) increases DNA synthesis and phosphorylation of p38 MAPK that, in turn, leads to cell arachidonic acid release, and production of TGF-$\beta_1$ and ECM proteins.

## LESSONS FROM GENE-TARGETED MODELS

The ACE2 gene knockout mouse may serve as a useful tool to investigate the effects of Ang-(1-7) on renal function. ACE2-deficient mice are viable, fertile, and are characterized by normal cardiac function and plasma levels of Ang II[20,66]. However, baseline blood pressures of ACE2-deficient mice vary according to genetic strain. In C57BL/6 mice, ACE2 deficiency is associated with a modest increase in blood pressure, whereas the absence of ACE2 has no effect on baseline blood pressures in 129/SvEv mice[20]. Deletion of the ACE2 gene in mice also causes late glomerulosclerosis and increased albuminuria[67]. These effects are reversed by treatment with the $AT_1$ receptor antagonist irbesartan, suggesting that ACE2 deficiency causes renal injury via impaired degradation of Ang II and subsequent activation of $AT_1$ receptors. However, the potential independent effect of reduced Ang-(1-7) generation on renal function in the ACE2 knockout mice has not been studied.

In this regard, the generation and characterization of Mas gene knockout mice has provided important clues on the potential role of Ang-(1-7) in normal physiology. The phenotype of the Mas knockout mouse appears to be highly dependent on strain. Mas-deficient mice on the mixed 129XC57BL/6 genetic background are healthy, grow normally, and show no obvious developmental abnormalities[68]. Indeed, these mice are normotensive and display normal plasma levels of Ang II[68]. However, Mas knockout mice on a pure C57BL/6 genetic background exhibit impaired cardiac function that is partially due to an increase in collagen expression to a profibrotic phenotype[69]. In addition, C57BL/6 Mas knockout mice exhibit a renal phenotype that is characterized by sodium and water retention, glomerular hyperfiltration, microalbuminuria, and renal fibrosis[70]. Interestingly, the renal phenotype of the Mas knockout mouse is associated with up-regulation of renal $AT_1$ receptors. Thus, it is possible that the phenotype of the Mas gene knockout mouse may at least partly be due to increased $AT_1$ receptor signaling activity, in addition to loss of Ang-(1-7) action. In contrast to these studies, Esteban et al. recently reported that C57BL/6 mice with Mas gene deletion had attenuated renal injury in the unilateral ureteral obstruction (UUO) and renal ischemia/reperfusion (I/R) models of renal injury[71]. Thus, Mas deficiency resulted in inhibition of NF-κB activation and reduced levels of cytokines, suggesting that blockade of Mas signaling events may prevent renal inflammation.

Mas-deficient mice on the FVB/N genetic background display a cardiovascular phenotype that is characterized by increased arterial blood pressure, lower eNOS expression, decreased NO production, and impaired endothelial function, compared to wild-type mice[72]. In addition, the NADPH oxidase catalytic subunit gp91(phox) protein is expressed at a higher level in Mas-deficient mice compared to wild-type, whereas superoxide dismutase and catalase activities are reduced[72]. Taken together, these data suggest that Ang-(1-7)–dependent signaling events that are mediated by Mas receptors may exert cardiovascular and renal protection by regulating blood pressure, limiting production of reactive oxygen species, and preventing the synthesis of ECM proteins. However, Mas-dependent signaling events appear to promote renal inflammation and thus could potentially exacerbate the progression of renal injury.

## ANG-(1-7) AND DIABETIC NEPHROPATHY

Diabetic nephropathy is the most common cause of end-stage renal disease, and is associated with activation of the intrarenal RAS and increased production of intrarenal Ang II, which mediates progressive nephron injury via the promotion of growth factors, inflammatory cytokines, and glomerular and interstitial fibrosis[73,74]. The effect of ACE2 on the development of diabetic nephropathy has been the subject of several studies and data support a renoprotective role. Renal expression of ACE2 is down-regulated in murine models of diabetes and in diabetic patients[19,21,75,76,77]. Pharmacological inhibition of ACE2 in streptozotocin (STZ)-induced diabetic mice increases albuminuria and glomerular ECM expansion[78]. These effects are associated with increased ACE expression in glomeruli and renal vessels. Thus, in diabetic nephropathy, the combination of increased ACE expression and inhibition of ACE2 could augment Ang II production, leading to accelerated glomerular injury. Similarly, Wong et al.

demonstrated that genetic deletion of the ACE2 gene accelerates diabetic renal injury[79]. ACE2 knockout mice were crossed with Akita mice, a model of type I diabetes mellitus. Diabetic ACE2 knockout mice ($Ace2^{-/y}Ins2^{WT/C96Y}$) exhibit increased albuminuria, mesangial matrix scores, and glomerular basement membrane thickening compared to diabetic control mice. Treatment of diabetic ACE2 knockout mice with the $AT_1$ receptor antagonist irbesartan reduced albuminuria. Therefore, a deficiency in renal ACE2 activity may accelerate diabetic kidney injury via impaired degradation of Ang II and increased activation of $AT_1$ receptors. However, the independent role of impaired Ang-(1-7) generation in contributing to the phenotype in diabetic ACE2 knockout mice remains unclear.

In this regard, Benter et al. reported a renoprotective role for Ang-(1-7) in the development of diabetic nephropathy[80,81]. Thus, administration of Ang-(1-7) to STZ-induced diabetic male rats reduced proteinuria and restored vascular reactivity in isolated renal artery segments[81]. Similarly, treatment of STZ-induced diabetic SHR with Ang-(1-7) attenuated NAPDH oxidase activation, diminished proteinuria, and decreased the diabetes-induced increase in renal vascular responsiveness to endothelin-1, norepinephrine, and Ang II[80].

In contrast, Shao et al. showed that chronic infusion of Ang-(1-7) to STZ-diabetic male rats accelerates renal injury[82]. Ang-(1-7) increased proteinuria as well as TGF-β1 mRNA and protein levels in the diabetic kidney, when compared to untreated diabetic rats. It is possible that differences in rodent strain and age, as well as the dose of Ang-(1-7) and length of treatment, could contribute to the discrepancies between these studies. Moreover, the cell-specific signaling pathways associated with Ang-(1-7) in the kidney could play a role in this variable response.

## ANG-(1-7) AND HYPERTENSION

In male SHR treated with the NO synthesis inhibitor NG-nitro-L-arginine methyl ester (L-NAME), Ang-(1-7) attenuates the elevation in mean arterial pressure[80]. This response is prevented by coadministration of indomethacin, a nonselective inhibitor of cyclo-oxygenase 1 and 2, suggesting that Ang-(1-7) may prevent development of hypertension via a prostaglandin-dependent vasodilatory pathway. In contrast, in rats with DOCA-induced hypertension, chronic infusion of Ang-(1-7) had no effect on blood pressure or cardiac hypertrophy , but significantly reduced collagen deposition in the heart[61]. Similarly, in rats chronically infused with Ang II, Ang-(1-7) infusion reduced myocyte hypertrophy and myocardial fibrosis, without significantly affecting blood pressure[59]. Thus, Ang-(1-7) appears to induce cardiac remodeling changes independent of effects on blood pressure.

Gender may play a key role in determining the renal effects of Ang-(1-7) in hypertension. In female Sprague-Dawley rats, Ji et al. utilized the renal wrap (RW) model of hypertension to investigate the role of Ang-(1-7) in the progression of hypertensive renal disease[83]. In RW ovariectomized (RW-OVX) rats, renal cortical ACE2 activity and protein were down-regulated by 31 and 30%, respectively. These effects were prevented by treatment with estrogens. In addition, RW-OVX rats exhibited reduced ACE2 activity and greater tubulointerstitial fibrosis and glomerulosclerosis than RW rats. Infusion of Ang-(1-7) into RW-OVX rats prevented the exacerbating effect of ovariectomy on the degree of renal injury induced by renal wrap hypertension. Importantly, the renal protective effects associated with infusion of Ang-(1-7) were not attributable to changes in mean arterial pressure, heart rate, body weight, or ACE2 activity. Ovariectomy increases the activity of NADPH oxidase in RW hypertension and Ang-(1-7) inhibits renal NAPDH oxidase activity in diabetic SHR[80]. Thus, Ang-(1-7) may prevent renal injury by attenuating renal superoxide formation induced by ovariectomy. These findings suggest that estrogens cause up-regulation of renal ACE2 and that increased intrarenal synthesis of Ang-(1-7) may protect against hypertensive renal disease. In the congenic mRen2 rat, Pendergrass et al. showed that females have higher ratios of renal Ang-(1-7) to Ang II compared to males, suggesting that sex differences in the renal RAS could account for the higher blood pressures in males[6].

In both rats and humans, chronic treatment with either ACE inhibitors or $AT_1$ receptor blockers increases plasma levels of Ang-(1-7) (5- to 25-fold)[84,85,86,87]. ACE inhibition contributes to increased plasma levels of Ang-(1-7) via increased availability of Ang I and inhibition of Ang-(1-7) metabolism. Antagonism of the $AT_1$ receptor increases Ang-(1-7) levels, presumably by increasing plasma levels of Ang II, which can be converted to Ang-(1-7) by ACE2.

This observation raises the possibility that Ang-(1-7) may contribute to the antihypertensive effects associated with ACE inhibition or $AT_1$ receptor blockade. In SHR, coadministration of Ang-(1-7) with the $AT_1$ receptor antagonist candesartan caused a marked reduction in mean arterial pressure[88]. This effect was reversed by $AT_2$ receptor blockade, suggesting that $AT_1$ receptor antagonism uncovers an $AT_2$ receptor–mediated vasodilatory response to Ang-(1-7). In SHR treated with lisinopril and losartan, Iyer et al. showed that administration of a monoclonal antibody against Ang-(1-7) caused significant elevations in mean arterial pressure, suggesting that Ang-(1-7) contributes to the antihypertensive effect of combined ACE inhibition and $AT_1$ receptor blockade[89]. In SHR, acute inhibition of Ang-(1-7) formation (via the use of two different NEP inhibitors) increased blood pressures approximately 20% above baseline, suggesting that Ang-(1-7) exerts a vasodilatory effect[90].

Increased ACE2 activity and subsequent formation of Ang-(1-7) may protect against the development of hypertension. Rentzsch et al. generated transgenic rats (TGR[SM22ACE2]) on a spontaneously hypertensive stroke-prone (SHRSP) genetic background that expressed the human ACE2 gene in vascular smooth muscle cells[91]. TGR(SM22ACE2) rats are characterized by increased plasma levels of Ang-(1-7), reduced mean arterial pressure, and a blunted vasoconstrictive response following intra-arterial administration of Ang II, compared to SHRSP control rats. Furthermore, TGR(SM22ACE2) rats exhibit an improved vasodilatory response to acetylcholine, compared to SHR-SP control rats. These data suggest that vascular ACE2 overexpression in SHRSP rats reduces hypertension and improves endothelial function, perhaps via the ability of ACE2 to metabolize Ang II to Ang-(1-7).

## CONCLUSION

The discovery of ACE2 and the generation of gene knockouts for this enzyme and for the receptor Mas have led to renewed interest in the role of Ang-(1-7) in renal pathophysiology. Ang-(1-7) regulates renal vascular tone and tubular transport, and affects signaling and growth in a cell-specific fashion. While evidence suggests that Ang-(1-7) is an endogenous vasodilator and may be protective in hypertensive renal injury, further studies are required to determine the impact of Ang-(1-7) on glomerular and tubular function in chronic pathophysiologic states, such as diabetic nephropathy.

## ACKNOWLEDGMENTS

This work was supported by grants from the Canadian Institutes of Health Research and the Kidney Foundation of Canada (to KDB), and an Ontario Graduate Student Scholarship (to MD).
